# A 1-Tesla MRI system for dedicated brain imaging in the neonatal intensive care unit

**DOI:** 10.3389/fnins.2023.1132173

**Published:** 2023-02-10

**Authors:** Elisa R. Berson, Ali Mozayan, Steven Peterec, Sarah N. Taylor, Nigel S. Bamford, Laura R. Ment, Erin Rowe, Sean Lisse, Lauren Ehrlich, Cicero T. Silva, T. Rob Goodman, Seyedmehdi Payabvash

**Affiliations:** ^1^Department of Radiology and Biomedical Imaging, Yale School of Medicine, New Haven, CT, United States; ^2^Department of Pediatrics, Yale School of Medicine, New Haven, CT, United States; ^3^Department of Neurology, Yale School of Medicine, New Haven, CT, United States

**Keywords:** Embrace^®^, point-of-care MRI, neonatal intensive care unit, brain imaging, 1-Tesla, hemorrhage

## Abstract

**Objective:**

To assess the feasibility of a point-of-care 1-Tesla MRI for identification of intracranial pathologies within neonatal intensive care units (NICUs).

**Methods:**

Clinical findings and point-of-care 1-Tesla MRI imaging findings of NICU patients (1/2021 to 6/2022) were evaluated and compared with other imaging modalities when available.

**Results:**

A total of 60 infants had point-of-care 1-Tesla MRI; one scan was incompletely terminated due to motion. The average gestational age at scan time was 38.5 ± 2.3 weeks. Transcranial ultrasound (*n* = 46), 3-Tesla MRI (*n* = 3), or both (*n* = 4) were available for comparison in 53 (88%) infants. The most common indications for point-of-care 1-Tesla MRI were term corrected age scan for extremely preterm neonates (born at greater than 28 weeks gestation age, 42%), intraventricular hemorrhage (IVH) follow-up (33%), and suspected hypoxic injury (18%). The point-of-care 1-Tesla scan could identify ischemic lesions in two infants with suspected hypoxic injury, confirmed by follow-up 3-Tesla MRI. Using 3-Tesla MRI, two lesions were identified that were not visualized on point-of-care 1-Tesla scan: (1) punctate parenchymal injury versus microhemorrhage; and (2) small layering IVH in an incomplete point-of-care 1-Tesla MRI with only DWI/ADC series, but detectable on the follow-up 3-Tesla ADC series. However, point-of-care 1-Tesla MRI could identify parenchymal microhemorrhages, which were not visualized on ultrasound.

**Conclusion:**

Although limited by field strength, pulse sequences, and patient weight (4.5 kg)/head circumference (38 cm) restrictions, the Embrace^®^ point-of-care 1-Tesla MRI can identify clinically relevant intracranial pathologies in infants within a NICU setting.

## 1. Introduction

Transcranial ultrasound is the most commonly used modality for evaluating brain structures and intracranial pathologies within the neonatal intensive care unit (NICU) setting (Pollatou et al., 2022). Over the past two decades, MRI has been increasingly used in neonates and infants to elucidate patterns of brain development (Damaraju et al., 2014; Kline et al., 2021; Zhang et al., 2021) and to quickly and accurately diagnose suspected intraparenchymal pathologies, such as ischemia and hemorrhage (Reddy, 2022). While transcranial ultrasound can reliably identify intraventricular hemorrhage (IVH) and ventriculomegaly, brain MRI is considered more sensitive in detecting white matter (WM) disease, especially acute ischemic injury (Nowell et al., 1988; Doria et al., 2014; Simonsen et al., 2015). According to 2020 “Routine Neuroimaging of the Preterm Brain” guidance from the American Academy of Pediatrics, brain “MRI for infants born at less than 30 weeks of gestational age is not indicated as a routine procedure” (Hand et al., 2020; Inder et al., 2021). However, there is an increasing demand for readily accessible and safe brain MRI neuroimaging in this vulnerable population.

MRI in very preterm infants (VPIs), defined as being born before 32 weeks gestation, is enabled by specialized protocols that reduce noise, minimize transport burden, and utilize smaller head coils (Dalal et al., 2006; Rona et al., 2010; Flick et al., 2011; Wang et al., 2014; Ghotra et al., 2021). A novel MRI technology that received Food and Drug Administration (FDA) approval in 2017 is the 1-Tesla Embrace^®^ for point-of-care brain MRI in the NICU (Voelker, 2017). Traditionally, MRI has been chiefly performed in term-equivalent infants stable enough for transport to the MRI suite. However, point-of-care 1-Tesla MRI enables safe imaging of a neonate’s brain in the NICU without the need to transfer. This technology with a permanent magnet, 150 mT/m peak gradient, and temperature-controlled bassinet can potentially allow for earlier confirmation and prognostication of IVH, ischemic injury, and periventricular WM changes among neonates who were traditionally only suitable for transcranial ultrasound (Goeral et al., 2021).

To date, in-NICU point-of-care 1-Tesla brain MRI scanners are only available in a few centers in the United States, with limited reports on comparisons of the point-of-care 1-Tesla brain MRI with transcranial ultrasound and conventional 3-Tesla MRI (Thiim et al., 2022). In our present study, we report the results from neonatal brain MRIs performed using a point-of-care 1-Tesla MRI system during our NICU’s first year of use. We compared intracranial findings on point-of-care MRI scans with conventional 3-Tesla MRI and transcranial ultrasound of neonates, whenever available.

## 2. Materials and methods

### 2.1. Patient characteristics

We retrospectively reviewed and evaluated the clinical and imaging information of all neonates who underwent a point-of-care 1-Tesla brain MRI from January 2021 through June 2022. The scanner can accommodate infants weighing up to 4.5 kg or with a head circumference of up to 38 cm. During this time, all clinically indicated non-contrast brain MRIs of neonates who fulfilled the above-mentioned physical criteria were performed in the point-of-care 1-Tesla brain MRI scanner, except for dedicated epilepsy protocol and neonates requiring contrast-enhanced MRI, who were referred for conventional 3-Tesla scanner given the imaging limitations for those indications with Embrace^®^. The institutional review board approved the research protocol for this study and waived the need for informed consent, given the retrospective nature of our analysis.

### 2.2. Brain MRI protocol

The infants were scanned within the NICU using a dedicated point-of-care 1-Tesla Embrace^®^ MRI scanner (Aspect Imaging, Shoham, Israel). The MRI protocols on this unit are limited but include T1-weighted spin echo [slice thickness: 4 mm; repetition time (TR): 600 ms; echo time (TE): 11–12.5 ms; flip angle: 90°; field of view: 14 cm × 14 cm; and matrix: 200 × 200] in the axial and sagittal planes; T2-weighted fast spin echo (slice thickness: 4 mm; TR: 9,900–12,000 ms; TR: 130–155 ms; flip angle: 90°; field of view: 14 cm × 14 cm; and matrix 200 × 200) in axial, coronal, and sagittal planes; diffusion weighted imaging (DWI) fast spin echo (slice thickness: 4 mm; TR: 14,000–15,500 ms; TE: 127–133 ms; *b* = 0 and 1,000 s/mm^2^; flip angle: 90°; field of view: 11 cm × 14 cm; and matrix 92 × 43) in the axial plane; and a 3D T1 gradient echo (slice thickness: 1 mm; TR: 20 ms; TE: 3.4 ms; flip angle: 15°; field of view: 14 cm × 14 cm; and matrix 140 × 140) acquired in the sagittal plane and reconstructed in axial and coronal planes. Additional apparent diffusion coefficient (ADC) and exponential DWI maps were generated from DWI acquisition. The total scan time for this protocol is approximately 45 min.

### 2.3. Comparison transcranial ultrasound and 3-T brain MRI

Most infants undergoing point-of-care 1-Tesla brain MRI also underwent additional imaging in the form of transcranial ultrasound or conventional 3-Tesla brain MRI. The transcranial ultrasounds were performed using 5–12 MHz phased array transducer (Phillips, USA) *via* anterior fontanel (coronal, sagittal planes) and mastoid (coronal plane) windows. The 3-Tesla brain MRIs were performed on a Skyra scanner (Siemens, Germany) in the radiology department.

### 2.4. Assessment of study findings and concordance

In addition to the official clinical report, all scans were re-reviewed by a neuroradiologist with over six years of experience in pediatric neuroimaging. To facilitate the presentation of findings, we summarized the exam indication into four main categories: evaluation of potential ischemic injury, intraventricular/intraparenchymal hemorrhage, term age scan in extremely preterm infants (<28 weeks gestational age), and other indications. The findings from each scan were corroborated with the official clinical report. Concordance was determined in comparison with the 3-Tesla brain MRI or transcranial ultrasound performed at the closest interval to the target point-of-care 1-Tesla MRI scan.

### 2.5. Statistics

The data are expressed as mean ± SD, frequency (percentage), median (interquartile), or ratios, wherever appropriate.

## 3. Results

### 3.1. Patient demographics

A total of 60 neonates were scanned using the point-of-care 1-Tesla MRI scanner. One MRI was prematurely terminated due to motion but was still included in our evaluation. Neonate demographics and clinical information are summarized in Table 1. Of these, 3 (5%) had 3-Tesla MRI, 46 (76%) had transcranial ultrasound, and 4 (7%) had both ultrasound and 3-Tesla MRI in addition to 1-Tesla scan. However, 7 (12%) patients had no other brain imaging available for comparison with the 1-Tesla scan.

**TABLE 1:** Demographic characteristics of infants who had 1-Tesla brain MRI.

Infants’ characteristics	*N* = 60
Gestational age at birth (weeks, mean ± SD, range)	30.4 ± 5.4 (22–40)
≤32 weeks	41 (68%)
32–36 weeks 6 days	7 (12%)
≥37 weeks	12 (20%)
**Method of delivery**	
Vaginal delivery	25 (42%)
Cesarean section	35 (58%)
Birth weight (grams, mean ± SD)	1,575 ± 1,040
**Apgar score [median, interquartile range]**	
1 min	5 [3–7]
5 min	8 [6–9]
10 min (only recorded for 22 patients)	7.5 [7–9]
Cord pH (only measured in 25 patients)	7.26 ± 0.14
Required respiratory support	35 (58%)
Seizures	4 (7%)
Initial blood glucose at birth (mg/dl)	72.8 ± 36.3
Initial hematocrit at birth	40.6 ± 9.7
Gestational age at time of 1-Tesla scan (weeks, mean ± SD, range)	38.5 ± 2.3 (29.9–43.6)
Days of life first 1-Tesla performed (mean ± SD)	56.3 ± 39.1
Days from 1-Tesla to nearest MRI or ultrasound (mean ± SD)	44.9 ± 33.2
**Primary indication for neonatal brain MRI**	
Routine pre-discharge scan in extremely preterm neonates	25 (42%)
Intraventricular hemorrhage	20 (33%)
Hypoxic injury	11 (18%)
Other	4 (7%)

### 3.2. Suspected ischemic injury

A total of 11 (18%) infants underwent point-of-care 1-Tesla MRI for suspected ischemic injury; of whom, two had evidence of ischemic injury, one had evidence of IVH, and one had a subdural hematoma. Below is a summary of imaging findings from two of these seven patients. Two additional patients are listed in Table 2 under the subcategory of “Suspected ischemic injury.” In the remaining three subjects, the point-of-care 1-Tesla MRI scans were normal and concordant with findings of other imaging modalities (transcranial ultrasound in two and follow-up 3-Tesla MRIs in one).

**TABLE 2:** Descriptors of infants who underwent 1-Tesla brain MRI imaging.

Age after birth of 1-Tesla MRI	Indication for 1-Tesla MRI imaging	1-Tesla MRI figures	1-Tesla MRI findings	Other imaging figures	Other imaging findings
**Suspected ischemic injury**					
7 days	Decreased fetal movement and profound anemia (hemoglobin 3.6 g/dl)	Figures 7A, B	Possible punctate focus of microhemorrhage in the right caudothalamic groove	Figure 7C	Transcranial ultrasound performed on the first day after birth confirmed the possible microhemorrhage
11 days	Fever, neonatal sepsis, and oxygen desaturations due to decreased respiratory drive	Figures 8A–F	No evidence of ischemic changes but demonstrated a 0.7 cm subdural hematoma	N/A	N/A
**Other indications**					
9 days	Evaluation for brainstem abnormality given decreased vocal cord mobility on laryngoscopic exam and obstructive apnea	N/A	No structural or signal abnormality in the brain	N/A	N/A
5 days	Evaluation after subgaleal hematoma was found on transcranial ultrasound	N/A	Redemonstrated subgaleal hematoma with no intracranial abnormalities	N/A	3-Tesla MRI redemonstrated subgaleal hematoma with no intracranial abnormalities
3 days	Prenatal diagnosis of ventriculomegaly and Trisomy 21		Mild dilation of the lateral ventricles to 11 mm at the level of the atrium		Transcranial ultrasounds performed before and after the MRI confirmed the ventricular dilation

**FIGURE 1:**
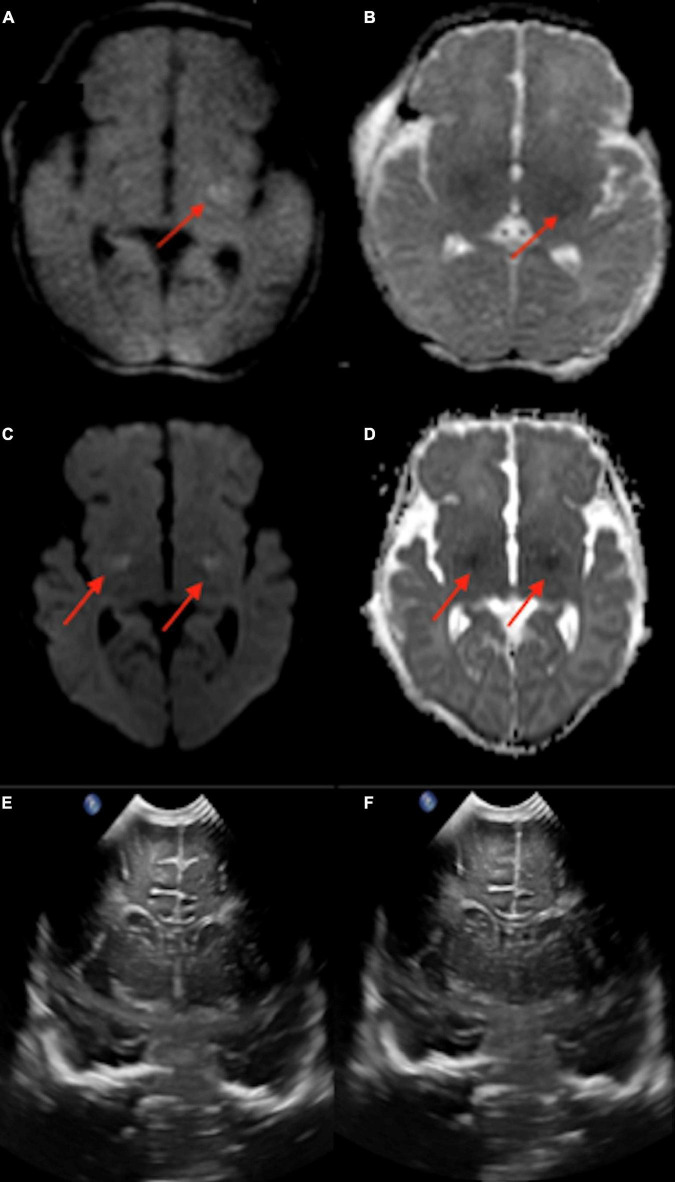
In a 4-day-old term neonate with challenging home birth, followed by transfer to hospital, resuscitation, and therapeutic hypothermia, the point-of-care 1-Tesla DWI **(A)** and ADC **(B)** showed reduced diffusion in the left basal ganglia. The next day (5 days old) follow-up 3-Tesla DWI **(C)** and ADC **(D)** showed ischemic changes and reduced diffusion in bilateral basal ganglia. Transcranial ultrasound was normal on the first day after birth **(E,F)**.

A 4-day-old infant, 40 weeks 5 days postmenstrual age (PMA), was born *via* home birth with subsequent difficulty breathing and probable seizure. The infant was resuscitated and treated with therapeutic hypothermia. The point-of-care 1-Tesla MRI showed reduced diffusion in the left basal ganglia (Figures 1A, B). The next day follow-up 3-Tesla MRI (5 days old) showed ischemic changes and reduced diffusion in bilateral basal ganglia (Figures 1C, D). Notably, the transcranial ultrasound on the first day after birth was normal (Figures 1E, F).

**FIGURE 2:**
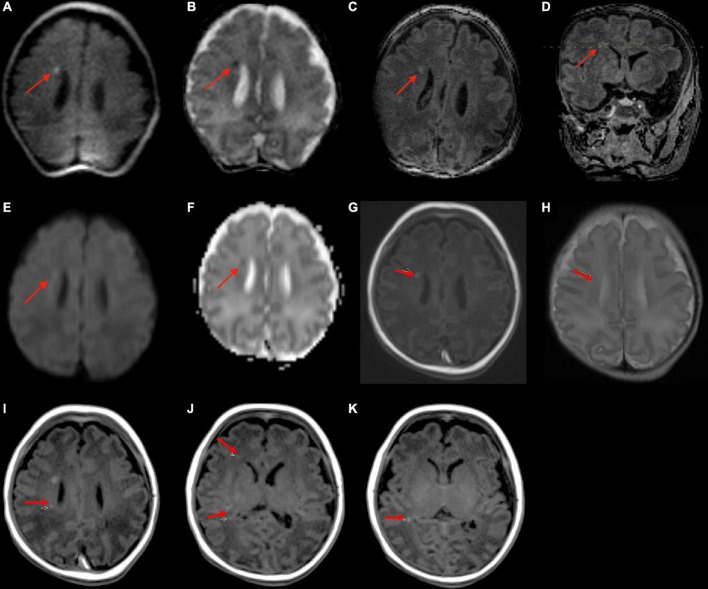
In a 5-day-old infant with a history of delivery complicated by shoulder dystocia requiring respiratory support and chest compressions, the point-of-care 1-Tesla DWI **(A)** and ADC **(B)** showed a punctate focus of reduced diffusion with associated T1 hyperintensity **(C,D)**. Two days later, follow-up 3-Tesla MRI showed diffusion restriction **(E,F)**, T1 hyperintensity **(G)**, and T2 hypointensity **(H)** of the same lesion. There were four additional punctate foci of parenchymal injury with intrinsic T1 hyperintensity on 3-Tesla MRI **(I–K)**, which could not be visualized on preceding day 1-Tesla scan. No transcranial ultrasound was obtained.

A 5-day-old infant (34 weeks and 2 days PMA) born to a COVID-positive mother with poorly controlled diabetes had delivery complicated by shoulder dystocia requiring respiratory support and chest compressions. The point-of-care 1-Tesla MRI showed a 4 mm focus of ischemic injury adjacent to the right caudate body (Figures 2A–K), which was confirmed on the follow-up 3-Tesla MRI on the seventh day after birth. Notably, four additional foci of punctate T1 hyperintensity were noted on the follow-up 3-Tesla MRI without corresponding signal abnormality on the initial point-of-care 1-Tesla scan (Figures 2I–K). The infant had no transcranial ultrasound for comparison.

### 3.3. Intraventricular/intraparenchymal hemorrhage

**FIGURE 3:**
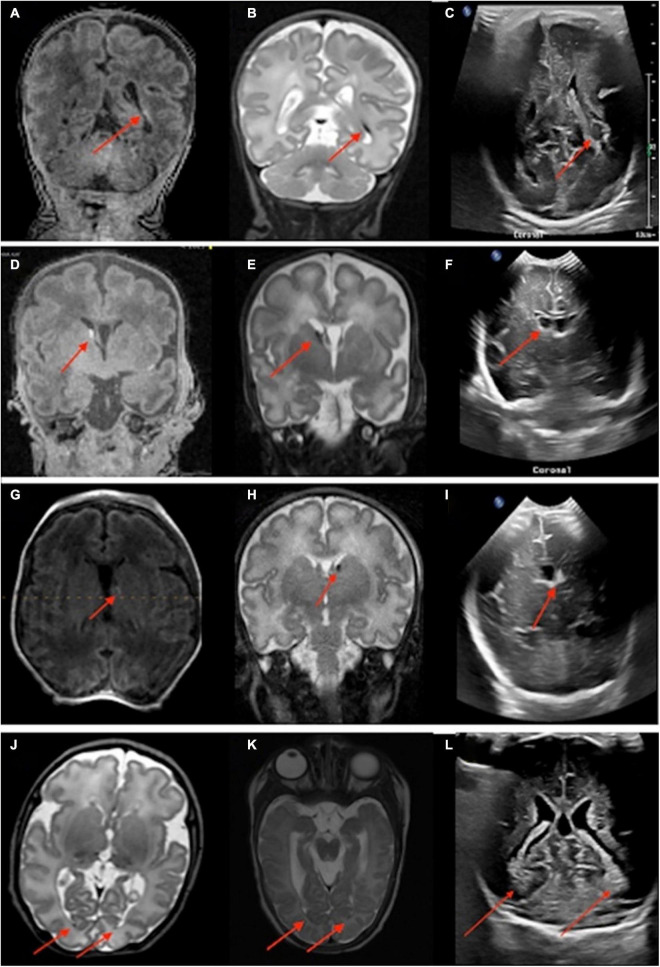
Neonates scanned on point-of-care 1-Tesla MRI for intraventricular hemorrhage (IVH) follow-up. An 80-day-old infant with T1 hyperintense **(A)** and T2 hypointense **(B)** grade II left germinal matrix hemorrhage on point-of-care 1-Tesla MRI performed 70 days after transcranial ultrasound **(C)**. A 52-day-old infant with T1 hyperintense **(D)** and T2 hypointense **(E)** right-sided grade I germinal matrix hemorrhage on point-of-care 1-Tesla MRI performed 34 days after transcranial ultrasound **(F)**. A 28-day-old infant with T1 hyperintense **(G)** and T2 hypointense **(H)** left-sided grade I germinal matrix hemorrhage on point-of-care 1-Tesla MRI performed 15 days after transcranial ultrasound **(I)**. An 85-day-old infant with layering T2 hypointense IVH **(J)** on point-of-care 1-Tesla MRI, which was also visualized on follow-up 3-Tesla MRI 38 days later **(K)** and was sequela of grade 2 IVH seen on transcranial ultrasound performed 74 days earlier **(L)**.

**FIGURE 4:**
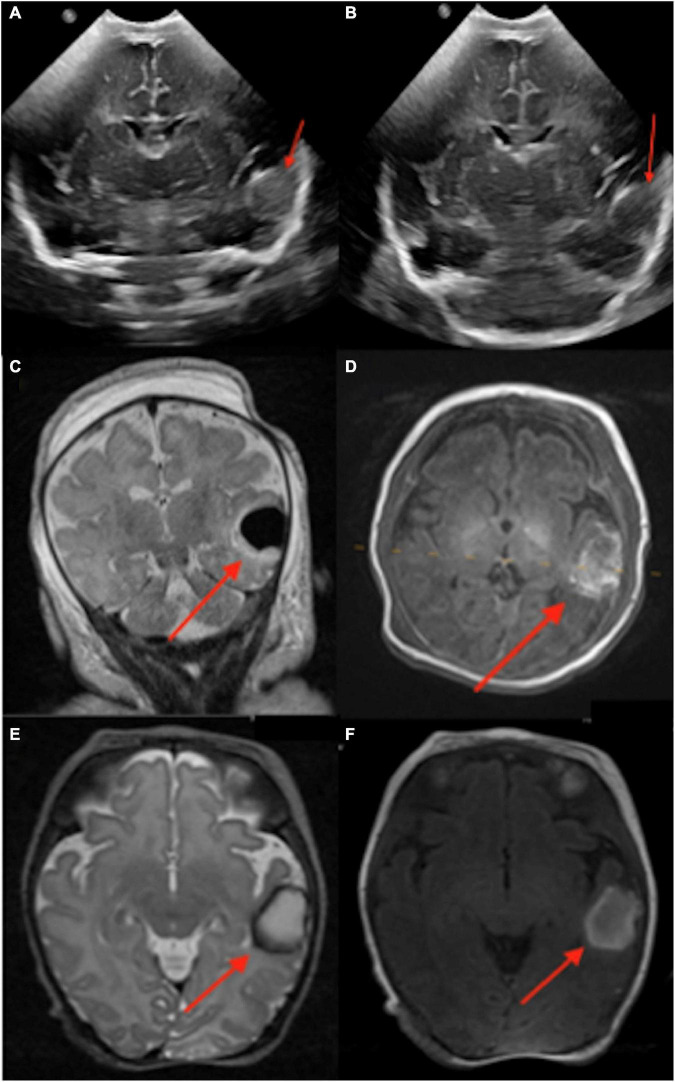
An infant born at 37 weeks and 3 days, with delivery complicated by shoulder dystocia and brachial plexus injury, was found to have hypoglycemia and seizures after birth. A left temporal lobe intraparenchymal hemorrhage was found in transcranial ultrasound (1 day old, **A,B**). This was further delineated on conventional 3-Tesla MRI (4 days old, **C,D**) and followed on point-of-care 1-Tesla MRI (35 days old, E,F).

A total of twenty infants were scanned at term age equivalent (36–40 weeks gestational age) or before discharge for follow-up of their IVH. In 17 out of 20 infants, IVH was found on point-of-care 1-Tesla MRI scan, corroborating the findings of the most recent preceding transcranial ultrasound. Images from four of these patients appear in Figures 3A–L. IVH findings were also confirmed in one infant on the follow-up 3-Tesla MRI (Figures 3J–L). In three infants with history of grade 1 or 2 IVH, there was no evidence of IVH on the point-of-care 1-Tesla MRI. This was concordant with the most recent preceding transcranial ultrasound for each patient performed prior to the MRI, all of which reported complete interval resolution of IVH. One infant who was born at 37 weeks and 3 days, with delivery complicated by shoulder dystocia and brachial plexus injury, hypoglycemia, and seizures shortly after birth, was found to have a left temporal lobe intraparenchymal hemorrhage on transcranial ultrasound performed at 1 day old (Figures 4A, B). Follow-up conventional 3-Tesla MRI performed on day four of life as part of an epilepsy protocol (Figures 4C, D), and a point-of-care 1-Tesla MRI (when 35 days old) delineated the extent of parenchymal hemorrhage (Figures 4E, F).

### 3.4. Routine pre-discharge scan in preterm infants

**FIGURE 5:**
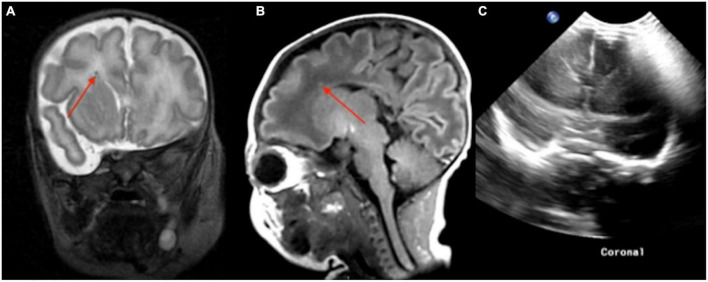
In a routine pre-discharge point-of-care 1-Tesla MRI of a preterm infant, there was a punctate focus of T1 hyperintense **(A)** and T2 hypointense **(B)** microbleed in the left anterior periventricular white matter which was not visualized on preceding transcranial ultrasound performed 5 weeks earlier **(C)**.

**FIGURE 6:**
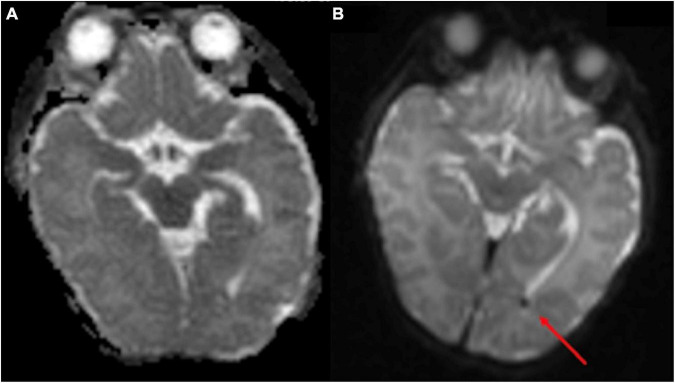
The point-of-care 1-Tesla routine pre-discharge MRI in a 4-month-old neonate was terminated after obtaining DWI/ADC due to infant movement **(A)**. Follow-up 3-Tesla MRI 5 days later showed layering IVH in occipital horns, which could be appreciated even on the ADC series **(B)**. No transcranial ultrasound was obtained.

A total of 22 infants with preterm birth had pre-discharge point-of-care 1-Tesla scans to exclude potential parenchymal injury. The postmenstrual gestational age at birth for this sub-cohort ranged from 22 weeks and 1 day to 34 weeks and 3 days. In a 63-day-old infant (36 weeks and 4 days PMA), the point-of-care 1-Tesla MRI scanner showed a punctate focus of microbleed in the deep WM next to the left frontal horn (Figures 5A, B), which was not identified on a preceding transcranial ultrasound performed 5 weeks earlier (Figure 5C). In a 4-month-old neonate (43 weeks and 4 days PMA), with no transcranial ultrasound, the pre-discharge point-of-care 1-Tesla MRI was terminated after obtaining DWI series due to infant movement (Figure 6A). However, a follow-up 3-Tesla MRI under sedation performed 5 days later showed layering IVH (Figure 6B), which was conspicuous on ADC series. This was not visualized on the ADC map from the point-of-care 1-Tesla MRI. In the remaining twenty infants, the point-of-care 1-Tesla brain MRI showed no structural or signal abnormality, concordant with transcranial ultrasound findings performed in 17 of these infants. In three infants, point-of-care 1-Tesla MRI was the only modality of brain imaging performed during the NICU admission at our institution.

### 3.5. Other indications

There were several other indications for the use of 1-Tesla MRI, including the assessment of brainstem abnormality, the evaluation of subgaleal hematoma, and a diagnosis of ventriculomegaly, which are detailed in Table 2 under the subheading of “Other indications.” Below are detailed examples of the evaluation performed for two infants with congenital fetal abnormalities. One infant was delivered *via* late preterm C-section due to a known omphalocele and was discovered to have a patent ductus arteriosus and patent foramen ovale. After surgical correction, the patient was intubated and placed on fentanyl and dexmedetomidine (Precedex) drips for 5 days. Term-equivalent MRI was performed on the 18th day after birth using the 1T MRI. The MRI excluded post-hypoxic sequelae or intracranial hemorrhage, concordant with an earlier transcranial ultrasound on the first day after birth.

Another infant with syndactyly of the bilateral second, third, and fourth digits in all extremities and two small appendages of the midline sacrum was scanned at term with 1T MRI. There was no evidence of any intrathecal abnormality on spinal ultrasound, and Embrace^®^ MRI performed on the third day after birth found no abnormalities.

## 4. Discussion

Our findings confirm the feasibility of the Embrace^®^ point-of-care 1-Tesla scanner for imaging in the NICU setting. Using this point-of-care 1-Tesla scanner, we could identify cerebral ischemic lesions in two of seven neonates with suspected hypoxic injury, which 3-Tesla MRI confirmed. Among infants scanned by the point-of-care 1-Tesla scanner, the only potentially missed lesions were punctate foci of parenchymal injury versus microhemorrhage (Figures 2I–K) in an infant with identified ischemic injury and small layering IVH in an incomplete study that only included DWI/ADC series (Figure 6). The point-of-care 1-Tesla MRI was more sensitive than transcranial ultrasound in detecting small parenchymal hemorrhage (Figure 5). This is especially important when considering small, difficult-to-detect lesions of the deep gray matter and brainstem. Of note, the implementation of Embrace^®^ point-of-care 1-Tesla scanner lacks a susceptivity-weighted imaging (SWI) sequence, although it is available in research protocols from outside the United States. In all infants with a history of IVH and intraparenchymal hemorrhage, point-of-care 1-Tesla MRI findings were concordant with the most recent transcranial ultrasound or follow-up MRI. Overall, the point-of-care 1-Tesla MRI could identify or exclude intracranial pathologies.

**FIGURE 7:**
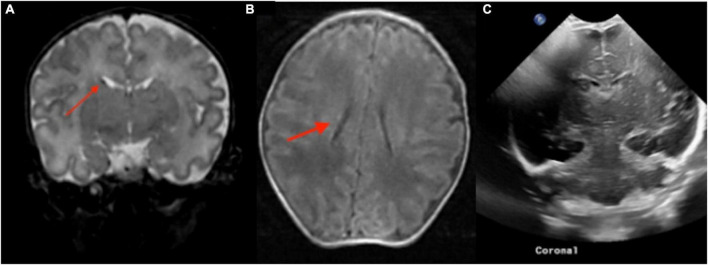
The point of care 1-Tesla MRI in a 6-day-old infant with emergency cesarean section delivery due to decreased fetal movement and variable decelerations showed a possible punctate focus of hemorrhage versus vessel in the right caudothalamic groove with T2 hypointensity **(A)** and T1 hyperintensity **(B)** but no evidence of ischemic injury. This was not seen on transcranial ultrasound on the first day after birth **(C)**.

**FIGURE 8:**
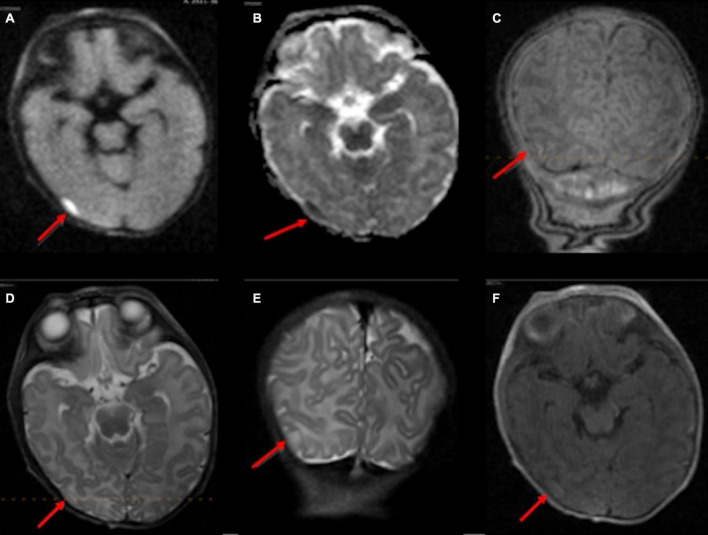
In an 11-day-old term neonate with a history of decreased respiratory drive, the 1-Tesla point-of-care MRI showed a subdural collection overlying the right temporo-occipital junction with reduced diffusion on DWI **(A)** and ADC **(B)**, which appeared isointense on T1 **(C,F)** and T2 **(D,E)**. No other brain imaging was performed.

Reliable detection of ischemic injury in an infant’s brain is especially valuable given transcranial ultrasound’s limitations in detecting early ischemic changes in cerebral parenchyma (Guan et al., 2017; Hwang et al., 2017; Salas et al., 2018). Among infants with point-of-care 1-Tesla MRI for suspected hypoxic-ischemic injury, we could identify parenchymal lesions in two infants, which were confirmed on follow-up 3-Tesla scans. In the remaining five infants, the point-of-care 1-Tesla could potentially identify microhemorrhage (not visualized on transcranial ultrasound, Figure 7) and subdural hematoma (Figure 8), while excluding ischemic parenchymal injury. However, the point-of-care 1-Tesla scan may be limited in detecting punctate foci of parenchymal injury (Figures 2I–K). In addition, the point-of-care 1-Tesla cannot currently obtain MRI angiography or venography series.

While transcranial ultrasound provides a readily available and sensitive tool for the evaluation of IVH and hydrocephalus in the NICU setting, MRI has the potential to increase the detection of intraparenchymal injuries, periventricular leukomalacia, and subdural hematomas (Inder et al., 1999; Williams and Hogg, 2000; Sie et al., 2005; Ahya and Suryawanshi, 2018; Arnold et al., 2023). A study by Rooks et al. (2008) found that transcranial ultrasound detected only 55% of infratentorial hematomas and no supratentorial subdural hematomas. In our series, the point-of-care 1-Tesla MRI could identify IVH in all neonates with such findings in their most recent transcranial ultrasound (Figure 3). It could also characterize subdural hematoma (Figure 8) and intraparenchymal hemorrhage (Figure 4). In two infants (Figures 5, 7), the point-of-care 1-Tesla MRI identified foci of intraparenchymal microbleeds that could not be detected on comparison transcranial ultrasounds. Of note, by adding an SWI sequence to Embrace^®^ scanners, the sensitivity of 1-Tesla MRI for detecting intraparenchymal hemorrhage is expected to increase in the future. In addition to a role in demonstrating intraparenchymal hemorrhage, neonatal MRI offers the opportunity to examine the effects of early anesthesia and more prolonged sedation on the brain of very young infants and to exclude associated central nervous system abnormalities in neonates presenting with axial or extra-axial deformities. Further studies with the 1-Tesla MRI can help to determine the potential clinical implications of the technology.

There is emerging evidence of clinical and research capabilities of point-of-care 1-Tesla brain MRI. Our results are in agreement with a recent report from Brigham and Women’s Hospital, which showed that Embrace^®^ 1-Tesla scanner could detect clinically relevant brain abnormalities in 207 scans of infants within the NICU setting (Thiim et al., 2022). In their series, 32 (15%) infants had a 3-Tesla MRI for comparison, and 80 (39%) had pathologies on their scans (Thiim et al., 2022), compared to our series, where 7 (12%) infants had a 3-Tesla scan comparison, and 33 (55%) had intracranial pathologies. A study from the Shaare Zedek Medical Center using the Embrace^®^ 1-Tesla scanner found that image quality was comparable to conventional 1.5 T MRI (Nun et al., 2022). Furthermore, using the Embrace^®^ 1-Tesla scanner, the institution demonstrated a positive association between increased T1 and T2 signal intensity in the basal ganglia and elevated serum bilirubin levels (Kasirer et al., 2021).

Aside from infants’ weight and head circumference restrictions, there are technical limitations in brain imaging with the Embrace^®^ 1-Tesla scanner. Many conventional MRI sequences are not currently available on the Embrace^®^ 1-Tesla scanner, most notably SWI, MRI angiography, or venography. The DWI sequence is also acquired using fast-spin echo instead of echo-planar imaging, which leads to a slightly longer acquisition time. Moreover, the magnetic field inhomogeneity is associated with distortion of images at the brain periphery and limits the reliability of evaluating calvarial, orbital, facial, or neck pathologies. There are also frequent DWI hyperintensity artifacts in the posterior aspect of the brain, which can be clinically resolved by examining exponential diffusion images. Finally, although acquiring post-contrast images on the point-of-care 1-Tesla scanner is possible, infants requiring post-contrast images were referred for 3-Tesla MRI.

Our study also has methodological design limitations to consider. It is retrospective, and not all infants with point-of-care 1-Tesla scans had a comparison 3-Tesla MRI or transcranial ultrasound. In addition, the comparison scans were at least 1 day apart from the target point-of-care 1-Tesla MRI, which is suboptimal given the dynamic nature of intracranial pathologies. Additionally, several imaging series in the point-of-care 1-Tesla scan were partially degraded by motion, despite using the specialized head coil and bassinet to restrict neonate movements. Lastly, this is a very early experience with evolving technology, and standardized indications and protocols at the study institution are continuing to develop. As a result, the potential of point-of-care 1-Tesla MRI in the NICU setting to contribute to the study of early brain development in healthy preterm infants or used as an adjunct in surveying for intracranial abnormalities in infants with other musculoskeletal, cardiac, or gastrointestinal abnormalities requires further exploration.

## 5. Conclusion

In a year-long experience at a single institution, we scanned 50 infants with Embrace^®^ point-of-care 1-Tesla MRI in the NICU setting and report this scanner’s feasibility in detecting clinically relevant intracranial pathologies. In comparing the point-of-care 1-Tesla MRI with other imaging modalities available in 45 (90%) infants, we demonstrated the advantages of point-of-care 1-Tesla brain MRI over ultrasound in detecting small early ischemic changes and parenchymal microhemorrhage. However, compared to the 3-Tesla scanner, tiny foci of parenchymal injury or IVH may not be conspicuous on point-of-care 1-Tesla MRI. Nevertheless, implementing a dedicated point-of-care 1-Tesla scanner in the NICU setting can make brain MRI accessible to very preterm and ill newborns at earlier ages while reducing risks of transportation and sedation. Early detection of clinically relevant intracranial pathologies, especially acute ischemic changes that are challenging to identify by transcranial ultrasound, has the potential to improve clinical treatment and facilitate early prognostication in the NICU setting.

## Data availability statement

The original contributions presented in this study are included in this article/supplementary material, further inquiries can be directed to the corresponding author.

## Ethics statement

Ethical review and approval was not required for the study on human participants in accordance with the local legislation and institutional requirements. Written informed consent from the participants’ legal guardian/next of kin was not required to participate in this study in accordance with the national legislation and the institutional requirements.

## Author contributions

EB, AM, and SPa made substantial contributions to the conception of the study, collected the images and clinical information of the patients, and were responsible for the analysis and interpretation of the data. EB, AM, SPe, ST, NB, LM, ER, SL, LE, CS, TG, and SPa were responsible for manuscript preparation, editing, and review and approved the version to be published. All authors contributed to the article and approved the submitted version.
